# “You hoped we would sleep walk into accepting the collection of our data”: controversies surrounding the UK care.data scheme and their wider relevance for biomedical research

**DOI:** 10.1007/s11019-015-9661-6

**Published:** 2015-08-18

**Authors:** Sigrid Sterckx, Vojin Rakic, Julian Cockbain, Pascal Borry

**Affiliations:** Department of Philosophy and Moral Sciences, Bioethics Institute Ghent, Ghent University, Blandijnberg 2, 9000 Ghent, Belgium; Center for the Study of Bioethics, University of Belgrade, Belgrade, Serbia; European Patent Attorney, Ghent, Belgium; Centre for Biomedical Ethics and Law, Faculty of Medicine, KU Leuven, Leuven, Belgium

**Keywords:** Autonomy, Big data, Care.data, Ethics, Health and Social Care Information Centre, Health research, Medical records, National Health Service England, Privacy, Trust

## Abstract

An ‘Information Centre’ has recently been established by law which has the power to collect, collate and provide access to *the medical information forall patients* treated by the National Health Service in England, whether in hospitals or by General Practitioners. This so-called ‘care.data’ scheme has given rise to major and ongoing controversies. We will sketch the background of the scheme and look at the responses it has elicited from citizens and medical professionals. In Autumn 2013, NHS England set up a care.data website where citizens could record their concerns regarding the collection of health-related data by the Information Centre. We have reviewed all the comments on this website up until June 2015. We have also analysed the readers’ comments on the coverage of the care.data scheme in one of the main national UK newspapers. When discussing the responses of citizens, we will make a distinction between the problems that citizens detect and the solutions they propose. The solutions that are being perceived as the most relevant ones can be summarized as follows: citizens wish to further the common good without being manipulated into doing it, while at the same time being safeguarded against various abuses. The issue of trust turns out to figure prominently. Our analysis of reactions to the scheme in no way pretends to be exhaustive, yet it provides various relevant insights into the concerns identified by citizens as well as medical professionals. These concerns, moreover, have a more general relevance in relation to other contexts of medical data-mining as well as biobank research. Our analysis also offers important pointers as to how those concerns might be addressed.

## Introduction

In 2012, the UK Parliament passed the Health and Social Care Act (HSCA). This law was introduced despite promises of the Conservative Party before the elections of 2010 not to carry out reforms of the UK’s National Health Service (NHS).^1^ One of the most significant changes resulting from the 2012 Act, which has been severely criticized, was the reduction of the Secretary of State for Health’s obligation to provide universal health care to UK citizens.^2^

Buried within this massive Act were provisions establishing the creation of an ‘Information Centre’, a body corporate with the power to collect, collate and provide access to the medical information for *all patients* treated by the NHS in England, whether in hospitals or by General Practitioners (GPs). Previously, i.e. before the enactment of the HSCA, patients’ hospital data had already been collected and made available to researchers and others by the NHS Information Centre (set up by the Health and Social Care Act 2010), the forerunner to the Information Centre (referred to by the NHS as the HSC Information Centre or HSCIC), and GPs had already started using standardised computerised record-keeping systems but these records were not transferred to a central database.

The report *Innovation Health and Wealth, Accelerating Adoption and Diffusion in the NHS*, known as the Carruthers report,^3^ discussed the uses to which the data collected by the new Information Centre might be put.^4^ This report provided indications that the hospital data have been used for purposes that are not strictly healthcare related (see below). With the background that there was concern that personal medical data might be used to individuals’ disadvantage or without due attention to maintaining confidentiality, a bill to amend the HSCA was brought forward in 2013 and signed into law in May 2014 as the Care Act 2014. This was none too soon as the harvesting of GP data by the Information Centre under the so-called ‘care.data’ scheme had been due to begin in Spring 2014.

The aim of this paper is threefold. First, we describe recent developments regarding the care.data scheme in the UK, including reactions from the medical community (which are discussed in the same section in view of the fact that they have had a clear impact on developments regarding the scheme). Second, we analyse and discuss concerns citizens have expressed regarding this scheme, on the NHS website as well as in one of the main national newspapers. Third, we relate these concerns to general discussions regarding governance and consent strategies that allow valuable research to proceed while still preserving the interests of research participants and the general public.

## Background of the care.data scheme and concerns expressed by the medical profession

On its website,^5^ the NHS states that it has developed the care.data programme as an initiative “to ensure that there is more rounded information available to citizens, patients, clinicians, researchers and the people that plan health and care services”, and “to ensure that the best possible evidence is available to improve the quality of care for all.” Among the benefits of the scheme are mentioned: the possibility for researchers to “identify patterns in disease and the most effective treatments”; the possibility “to find more effective ways of preventing or managing illnesses; advise local decision makers how best to meet the needs of local communities; promote public health by monitoring risks of disease spread; map out pathways of care to streamline inefficiencies and reduce waiting times; determine how to use NHS resources most fairly and efficiently.”

The potential positive impact of the care.data programme has been acknowledged by various organisations. The idea of better use of existing data was already advanced in 2013 in a joint statement of the Royal College of General Practitioners, the British Medical Association, NHS England and the HSCIC: “Greater transparency and better use of data to improve the quality of patient care are ambitions we can all support. Anyone making healthcare decisions needs access to high quality information: doctors need it to inform their clinical decision making; patients need it when deciding which treatment is best for them; and commissioners need it when making decisions about which services are right for their populations.”^6^ In a separate press release in December 2014, the Royal College of General Practioners acknowledged that: “care data is a vitally important project that has the capacity to bring enormous health benefits to patients up and down the country”.^7^

However, the scheme has met with a lot of opposition. In Autumn 2013, NHS England had set up a care.data website where citizens could record their concerns, and an information leaflet on care.data had been sent to all English households in early new year 2014. The leaflets resembled junk mail and were binned unread by many. However, public concern rose, as did concern among GPs. Many patients began to take advantage of the opportunity to opt out that the Secretary of State for Health had offered (even though the HSCA does not itself provide for the opportunity for opting out and there is no certainty that the offer might not quietly be withdrawn one day).

With many GPs showing concern, and with one Oxford-based GP in particular threatening to opt all his patients out of the scheme, the government decided to delay GP data harvesting until Autumn 2014 to allow NHS England the opportunity to persuade GPs, healthcare workers and patients that the care.data scheme was valuable and that sufficient safeguards had been put in place. Again, just in time since in June 2014 the Partridge Report^8^ on worrying data misuse by the forerunner to the HSCIC was published.^9^

Moreover, it was decided to begin GP data harvesting within a group of ‘pathfinder’ areas in England,^10^ and to initiate a series of public discussion sessions with the Care.data Advisory Group. A recent report suggests that data harvesting might begin as early as June 2015.^11^ An earlier report from the same source indicated that, initially at least, data would go not to the general research database of the HSCIC but to a ‘quarantined’ half-way house.^12^

GP support for the care.data scheme seems to be weak, with a report in August 2014 of a survey of GPs showing one-third likely to opt their patients out if NHS England rejects the call by the British Medical Association (BMA) for the scheme to be based on patients opting in:

Almost a third of GPs say they intend to opt their patients out of the care.data scheme if NHS England doesn’t accept calls for the scheme to be run on an opt-in basis.

Pulse’s survey of more than 400 GPs reveals that 31 % of respondents said they would opt patients out - despite this being unlawful - while only 32 % said they would not be opting patients out. …

Many GPs also said that inadequate safeguards and lack [of] clarity of where data can be shared and what it can be used for were behind their decision to opt patients out. …

… a GP in Crawley said that … : ‘Care.data is a fantastic research tool and used properly could help drive change that will benefit us all. The problem is that the central bureaucracy of the NHS has ignored the rights of individuals.’ …

… a GP in Ecclestone, Lancashire, said: ‘Initially most patients are willing to join the scheme as they feel it is a good idea if the emergency doctors knew about their medical conditions.’ ‘But once [we have] explained that their records could be seen by non-medical people and could be used for pharmaceutical research purposes, they seek to withdraw consent.’^13^

The BMA’s position on care.data, adopted at the Annual Representative Meeting in July 2014, is as follows:

That this Meeting agrees that the care.data system should not continue in its present form as:

i) it lacks confidentiality and there is a possibility for individual patient data to be identified;
ii) it carries the risk of GPs losing the trust of their patients who may feel constrained in confiding in them;
iii) the future potential users of the data are not well defined;
iv) it should be an opt-in system rather than an opt-out one;
v) the data should only be used for its stated purpose for improving patient care and not sold for profit.^14^

The *amendments* to the HSCA passed in the Care Act 2014 go some way towards addressing the concerns regarding confidentiality and inappropriate use of patient health data, not least by specifying in Section 122 that Section 261 HSCA is amended to allow data release only: ‘for the purposes of—the provision of health care or adult social care, or the promotion of health.’ However, the precise boundary imposed by this amendment is unclear. It seems clear that the amendment excludes making the data available to actuaries for the purpose of determining life insurance policy terms. However, it clearly does not prevent the data from being made available to drug researchers and pharmaceutical firms.

It is appropriate therefore to question just what information is to be harvested from patients’ GP records, and indeed whether a patient has any right to block such use. In this regard, the advice to GPs given by NHS England itself is of interest:

Under the Health and Social Care Act 2012, NHS England has the power to direct the HSCIC to collect information from all providers of NHS care, including general practices. … Guidance will support General Practices by explaining how patient information will be collected, anonymised and used by commissioners so they can better understand the true outcome of care provided to patients and continuously improve health services for all.

The General Practice Extraction Service (GPES) will be used to extract GP data each month. The identifiers to be extracted are: NHS number, date of birth, postcode, and gender which will allow patients’ GP data to be linked to their hospital data. No free text will be extracted, only coded information about referrals, NHS prescriptions and other clinical data.^15^

Moreover, the GP readers were directed to a blog, on NHS patient information and the Data Protection Act (DPA), of the Information Commissioner’s Office (ICO), i.e. the official body set up to enforce and oversee data related legislation. The ICO explains that:

GPs holding personal information about patients is nothing new and is covered squarely by the DPA. Generally everyone understands what’s happening: you give personal information to your GP who then records that information as your medical history. This record may include information from other health services and allows your GP to track your health throughout your lifetime.

The changes begin with some of the personal information included in that record going from GPs to the Information Centre. This happens under the direction of NHS England, which is allowed due to a new law, the Health and Social Care Act 2012.

This law gives NHS England the right to direct the Information Centre to collect certain sorts of data from the medical records. The law is a statutory enactment which requires the disclosure of the data, which means the data becomes exempt from the main parts of the DPA [Data Protection Act].

Because the main parts of the DPA are exempt it means that neither GPs (as data controller) or patients (as data subjects) have the right to stop that information being taken into the Information Centre – there is no legal ‘opt out’ under the DPA.

But while the DPA doesn’t give patients a right to object, the Secretary of State for Health has offered patients an option not to have their information used in this way. But as this option isn’t covered by the DPA, we can’t regulate it, and we don’t set the rules on how it works.^16^

As is clear, the option to opt out of care.data is not overseen by the Information Commissioner’s Office, and, as we noted earlier, this option is not guaranteed by law.

Moreover, the legal basis for the care.data scheme ‘trumps’ key provisions of the Data Protection Act.^17^ The 2012 Act (the HSCA) allows all patient data to be used for purposes that extend beyond patient care (e.g. for research) without any consultation, i.e. without the patients’ knowledge. Thus, the law *makes it impossible for patients to prevent their data from being used for research*. Yet under the Data Protection Act 1998 (S 2(1)(a), Part II, Schedule 1), any health professional gathering personal information directly from a patient has a responsibility to advise the patient of the intended uses of the information, unless this would be impracticable. As Jamie Grace and Mark Taylor have convincingly argued, in this regard the Data Protection Act may be overridden by the HSCA, since the direct recipient of the information from the patient, the patient’s physician, is *obliged* to forward such information to the HSCIC which itself is *notobligedto inform the patient* of the use of such data once ‘anonymised’.^18^ This has far-reaching consequences:

[T]he Information Centre will have the power, under Section 259 [of the HSCA], to *require* confidential patient information (and other information) from health and social care bodies…^19^ [A] disclosure to the Information Centre, in response to a *requirement* that it be provided,… will not constitute a breach of the common law duty of confidence and will satisfy the requirement that there is a lawful basis for the processing of sensitive personal data under Schedule 3 of the Data Protection Act 1998.^20^

Consequently, as observed by Grace and Taylor, the *right of a patient to object* to processing of his personal data on the basis that it would be likely to cause ‘substantial damage or substantial distress to him or to another, and that damage or distress is or would be unwarranted’ (*cf.* Section 10 of the Data Protection Act 1998) *is simplyremoved* as a result of the HSCA:

[T]he responsibility to consult the patient and provide her with the opportunity to object] is lifted in relation to *both* the Information Centre *and* health professionals *if disclosure of the information has been required by the Information Centre*.^21^

Following long-existing UK government practice in relation to governmental ICT schemes, both the HSCIC^22^ and NHS England^23^ have produced ‘Privacy Impact Assessments’ in relation to the potential impacts on patient privacy of the operations of the HSCIC and of the care.data scheme respectively. These are particularly of concern on two points—the extent to which citizens’ registered opt-outs will be honoured and the awareness of NHS England of ethical concerns extending beyond the potential for harm if medical confidentiality is breached. These Privacy Impact Assessments will be returned to below.

As shown in this section, key elements of the discussions regarding the care.data scheme are focused around the following topics: appropriate consent mechanisms for data collection and use; the right to object to processing of personal data; the extent of the data collected; and the uses of the data by the NHS or third parties. Various of those concerns were also raised by citizens, as we will see in the following section.

## Citizens’ reactions to care.data

As mentioned earlier, in Autumn 2013, NHS England set up a care.data website where citizens could record their concerns regarding the collection of health-related data by the HSCIC. Early in 2014, an information leaflet on care.data was sent to all English households.

In this Section, we will provide a few examples of the many comments that have been posted on the care.data website. We have reviewed all the comments on this website up until 1 June 2015.^24^ As of that date, the NHS England care.data blog contained 201 blog entries.^25^ From the blogger names given, 171 individual bloggers were responsible for the 201 entries. However, it cannot be ruled out that individual bloggers may have used more than one blogger name.

The analysis involved extracting from the 201 entries the separate points of concern raised by the bloggers and subsequently identifying generic headings under which these points of concern could be categorised. These generic categories were not identified in advance, but instead emerged from the analysis of the comments made by the bloggers. The method of thematic analysis was used, i.e. a qualitative research method for identifying, analysing and reporting themes within data.^26^ Hence the points of concern were not quantified, i.e. we did not determine which were most frequently or least frequently raised. The identification and analysis of themes was done independently by authors SS and JC. Any differences in categorisation or analysis were discussed by all the authors and consensus was always reached.

The purpose of our analysis was to identify the generic categories of citizens’ concerns from the blog entries and, subsequently, to compare those with the types of concern raised by medical professionals and by the NHS itself. To broaden the data set used for the analysis of citizens’ concerns, and to check whether there might be any further generic categories for points of concern amongst the public, it was decided to review the blog entries invited by one of the United Kingdom’s major and renowned national newspapers. The UK has four newspapers in this category, The Independent, The Times, The Daily Telegraph, and The Guardian. The Times was excluded because its blog is accessible only to subscribers. The Guardian was chosen because of its relatively extensive coverage of the care.data scheme. Two blogs from The Guardian were chosen.^27^ These blogs concerned articles which respectively might prompt concern and calm about the scheme. From the blogger names, which overlapped to some extent, it appeared that blog entries were made by a total of 85 individuals. It is quite possible that there was some overlap between the bloggers on The Guardian’s website and those on NHS England’s website. The blog entries were analysed in the same manner as those for the NHS England care.data blog but with the generic categories identified from the NHS England care.data blog already in place. No new generic categories were found.

To be clear, we do not imply that the comments in these three blogs are representative of the UK population. The bloggers are a sample of self-selected citizens who have voiced concerns regarding the sharing of health data. However, their comments do give us very clear and useful pointers as to the concerns that figure prominently within the population. Although the UK is of course only one case, the issues that have been raised by the bloggers are highly relevant and can serve as an excellent basis for future investigations of the issue of health data sharing.

Seven major concerns were raised by citizens about the care.data scheme: lack of transparency; lack of respect for confidentiality and privacy; misgivings about the opt-out scheme; erosion of trust in GPs and the health care system; wrongful appropriation of personal property; commercialisation; and uses of personal health data that conflict with the person’s moral values.

### Lack of transparency

First, various citizens point to a lack of transparency and complain that the care.data scheme has been insufficiently advertised or even advertised in a way designed to be misleading. The belief that they lack sufficient information, frequently augmented by the suspicion that information is being withheld on purpose, is clearly mirrored in the following statements of citizens:

This NHS datacare leaflet was dropped through my door with a take-away promotional leaflet so I nearly threw them both away by mistake. Perhaps that is exactly what the NHS hopes will happen. Care.data, 14.01.14

You couldn’t have made the leaflet look more like junk mail if you’d tried; I assume that was deliberate so that people wouldn’t know about it and therefore wouldn’t opt out. Care.data, 30.01.14

The lack of publicity about the biggest change in the relationship between GP and patients is little short of a national disgrace. Care.data, 17.02.14

I have no particular problem with the general idea of data sharing, but I am greatly concerned about the underhand way in which this is being done. Like (apparently) the majority here my husband and I received [sic] no notification of this, nor were there public messages informing the public that this information was coming (or supposed to be). Care.data, 20.02.14

No information has been circulated - even the local GP surgery knows nothing and has no information. Come on - this is a disaster. I refuse to accept that a serious effort was made to inform the public - rather you hoped we would sleep walk into accepting the collection of our data. Care.data, 19.02.14

### Lack of respect for confidentiality and privacy

Second, various citizens express the fear that personal health data might be accessible to and/or obtained by others, particularly insurance companies, employers and recruitment agencies. The following quotes illustrate this fear of a lack of respect for confidentiality and privacy.

It is a total disregard of our privacy, Care.data, 13.01.14

Despite all the assurances here and elsewhere about privacy and security, I have absolutely no faith that my data will be secure. There have been numerous recent instances of data loss, hacking and other breaches of supposedly secure government networks (pensions, benefits and records armed-service personnel, for instance). And those are just the mishaps they haven’t been able to cover up. I see no reason to believe that this new database will be any more secure. Care.data, 20.01.14

I am concerned that even anonymised information could be combined with other information that’s easily available to de-anonymise and identify me. I’m also concerned that other moves that are planned for the future will further erode patient confidentiality beyond what has already been published. Care.data, 04.02.14

As I appreciate it, the amount of detail included within one’s personal records would easily allow direct identification of individuals. Allowing such full personal information to go outside the NHS will enable commercial organisations to target individuals. It may take time for the risks to become appreciated but information will gradually migrate outwards into possibly unscrupulous hands. Care.data, 13.02.14

The care.data/HSCIC health record data extract seems to drive a coach and horses through the Data Protection Act and the Human Rights laws. Care.data, 12.02.14

There are very good reasons to fear that so called secure systems will be breached, either through incompetence or special interests, as they have in the past. I do not believe that my information will be kept secure and therefore do not trust this system. I forbid this sharing of my personal data. I did not sign up for dissemination of my health records. The NHS may regard me as just a statistic, but that is not the point of my participation in public health care. Care.data, 20.10.14

Drunk driving’s still an offence even if you don’t have an accident, because society thinks the potential of harm is sufficient. Are you seriously saying that medical confidentiality only matters in retrospect if the failure causes explicit harm, rather the potential for harm? The Guardian, 19.08.14

### Misgivings about the opt-out scheme

Third, various citizens express strong misgivings about the opt-out scheme. Citizens express dissatisfaction that decisions about them are being taken without them being consulted or being able to consent. This is seen to apply not only to the individuals raising this concern but also to relatives who are unable to give consent, for example minors.

I object strongly to having to opt out of this rather than opt in. Care.data, 21.01.14

… How do newborns opt out? How do children opt out? How do the mentally disabled opt out? What if my partner and I can’t agree as to whether our children should be opted in or out? In out in out shake it all about? Care.data, 28.01.14

If care.data is so manifestly in the interests of all, why don’t you take the obvious ethical approach, and make it OPT-IN by default? Care.data, 22.02.14

I agree totally it should be an Opt in situation not opt out, surely they have to have permission? Care.data, 13.01.14

This is basically a decision made for me, without me. Care.data, 01.03.14

the need to actively opt out, and the absence of any official form for doing so is an absolute disgrace. Care.data, 20.01.14

I … object to the way in which they are making it hard for people to opt out - why is there no form for people to fill in and have forwarded to their own medical practice. Care.data, 24.01.14

There is no information on how to opt out, or even an opt out form. Care.data, 29.01.14

### Erosion of trust in GPs and the health care system

Fourth, various citizens point to an erosion of trust in GPs and the health care system. They fear that the care.data scheme will make it difficult or impossible for them to continue to trust their doctor and their healthcare system:

Patients will not confide in their doctor, certain personal information, that may be absolutely necessary for diagnoses and treatment, knowing that outside agencies may have access to it. Care.data, 26.02.14

It is a disgrace and will prevent patients being honest with their GP Care.data, 01.03.14

I do not trust the government with my data, and now I cannot trust my doctor o[r] the wider NHS. Care.data, 05.05.14

The NHS has to build trust, and it won’t do that with emotional blackmail and hysterical claims that *people will die if we can’t sell your data*. This didn’t work before, and it’s still nonsense now. The Guardian, 18.08.14 (emphasis in original)

I would welcome the good that could come from care.data and would welcome the gifting of information from individuals who make up the community for the common good. But they (care.data) blew it. They blew it by being patronizing and disingenuous and by being unlucky enough to be preceded by Wikileaks exposures. They need to regain trust by apologizing for their previous abject failure and then by persuading us as individuals that a properly anonymised, secure version is safe and effective. The Guardian, 18.08.14

They should have been more open about the users. They didn’t have adequate criteria to decide who should get the data. And I’d add this needs to be clarified further going forward, because the [2014] Care Act wording is not specific and not well communicated to the public. Perhaps most importantly I’d disagree with, “none of the uses count as causing harm to patients” yet the harm is already done, and you can’t measure it. People are withholding information from their GPs, and have lost trust how their data is used. There is harm caused by worry, which you can’t count. The Guardian, 18.08.14

Now [the scheme is] on indefinite hiatus with a shrinking number of GPs willing to take part in the pilot and the BMA demanding opt-in for everyone…. Some surgeries are reporting hundreds of written opt-outs. Would you say this has been a successful project so far? From where I’m sat it’s a complete shambles. …The NHS thought they could do this without consent. They were wrong. Simply shouting louder isn’t going to work, you need to engage and stop patronising us. The Guardian, 20.08.14

### Wrongful appropriation of personal property

Fifth, various citizens voiced the concern that personal health data and medical files are personal property and that the government does not have the right to appropriate such data under the care.data scheme. The following quotes highlight this concern:

The whole exercise is nothing more than this government selling off things which it does not own. Care.data, 31.01.14

My medical records - and those of my children - are my property. Neither the government nor the NHS has the right to sell [them] either for profit or for the advantage of private companies, business interests or political advantage. Care.data, 31.01.14

I would like to know why they want my information, what for, and who [it’s] likely to go to … It is polite to ask us[,] they are our records[, and] if the bank did this they would be fined. Care.data, 13.01.14

The NHS needs to think again on who owns medical data (we the patients do) Care.data, 29.01.14

### Commercialization

Sixth, various citizens express worries that personal health data will be sold to third parties, including commercial companies, and that commercial and for-profit activities will be developed on the basis of their personal health data. The following quotes reveal this concern:

… I’m outright fuming that a government organization like the NHS can sell my personal data without even asking for permission! Care.data, 23.02.14

Basically they have been selling it. Anything for a few quid. Are the British public now a commodity for this government … to sell? It will be tissue next. Guardian, 18.06.14

In the past 2 years, a range of researchers and private companies have applied and received sensitive medical information held by the NHS…. [W]ith the promise of a nation-wide data pool, private companies will be queuing to get their hands on medical records. Care.data, 16.01.14

Just wanted to add I would have considered this long and hard if only the NHS had access to these details… As soon as you add private companies to the equation it loses its validity. Care.data, 16.01.14

I am so angry about this I thought our records were private not up for auction to the highest bidder. Care.data, 18.02.14

### Uses of personal health data that conflict with the person’s moral values

Finally, some citizens expressed worries about the potential uses of personal health data for purposes that an individual might consider unethical or inappropriate or, put differently, uses of personal health data that conflict with the person’s moral values. For example:

While I may be willing to share all my data for the purposes of improving health for the world at large, I find the language used vague -most probably- on purpose. There is no guarantee that my data will be used ethically, All it says is that there are “strict rules to protect” privacy. I want strict rules to protect my data from being used in research relating to the creation, marketing or deployment of weapons; I want my data to be protected from being sold to or shared with companies which engage in the patenting of genome products; I want my data to be protected from being sold to or shared with companies that engage in abusive hiring practices here and abroad; I want my data to be protected from being sold or shared with companies or individuals that treat the environment with contempt; I want my data to be protected from being sold to or shared with companies that have unacceptable top executive salaries; and this is just a sample. I want the data to be supervised by an independent forum of individuals whose remit is to follow strict published ethical guidelines relating to sharing, selling and profit making by the use of my data. Care.data, 22.01.14

## Citizens’ views on how to move forward: promoting the common good and creating transparency

The various concerns identified show that many citizens are suspicious about how their data will be used. How will citizens’ privacy be protected? What role do GPs have in the possible misuse of data? Will governments misuse citizens’ health data? Will these data be commercialised? Will they be utilised with disregard for moral values? At the same time, the analysed data shows that many citizens would like to believe that sharing of health data may serve the common good. This suggests that a desire to be good to others, to be useful to others and to society, plays an important role in decisions about the sharing of health data. The following statements further hint at how important these citizens believe is that they further the common good. Expressions of concern for the “health of the world at large” clearly point in that direction. Here are a few statements illustrating this concern:

I do believe that there are potential benefits to data sharing in general, as long as it’s used by the right people for the right reasons. Care.data, 28.01.14

Consider questions like this[:] 1 - Does pill X lead to a greater incidence of stoke/heart problems/cancer etc. 2 - Does smoking with pill y lead to particular issues? 3 - Is a particular type of cancer more prevelant [sic] in a particular area? If so is there anything else in those peoples medical records to explain why? Questions of this type, and more, can be answered by analysing mass amounts of data, which isn’t available on this scale otherwise. It’s invaluable for research and will help improve the health of the nation. Care.data, 24.02.14

I do not want my data ending up in the hands of corporations whose profit focus undermines the aims and ethics of the NHS. Care.data, 28.02.14

A deliberate decision was made to sell our data to a private company for purely commercial use, having nothing whatsoever to do with improving medical care or NHS services. This government has lied about its intentions for the NHS, and continues to lie and obstruct information about what it has done with it, and what is going on as we speak. This is all really too bad, because the existence of a large database like the NHS has such obvious benefits. However this is only where the NHS remains a public service, and where the information is used to benefit the public. NOT commercial businesses. The Guardian, 18.08.14

In response to the lack of adequate information and transparency, many citizens plead for more and better information provision. Instead of a general leaflet, various citizens indicated the importance of being addressed personally about data sharing.

It is sad that such important information is distributed like this. This is so important for everyone to understand what is happening, that it should have been personally addressed to all. Care.data, 31.01.14

I strongly object to the way patients are actually being informed about this. Today I received the leaflet ‘Better information means better care’ together with a load of junk mail which I could have easily binned. I suspect many people will not give it a second look. There should have been some personal correspondence from one’s GP practice informing patients about this rather than a mailshot. Care.data, 21.01.14

## Ethical concerns raised in the Privacy Impact Assessments of the care.data scheme

As mentioned earlier, with regard to governmental ICT schemes, the UK government has a long tradition of commissioning so-called ‘Privacy Impact Assessments’. Thus, the HSCIC^28^ and NHS England^29^ have each produced a Privacy Impact Assessment in relation to the potential impacts on patient privacy of the operations of the HSCIC and of the care.data scheme respectively. The care.data Assessment produced by NHS England is particularly relevant for the purposes of this paper, for two reasons.

The first reason has to do with the extent to which the Assessment *acknowledges ethical concerns relating to the care.data scheme, several of which are clearly similar to citizens’ concerns discussed above*. This can be seen from the following passages:

The [HSCA] … sets aside the requirement under the common law duty of confidence to seek patient consent. … The extraction of personal confidential data from [health service] providers without consent carries the risk that patients may lose trust in the confidential nature of the health service. (page 6)

Some people may feel a loss of individual autonomy (no patient consent) … Some patients may not be aware of or understand their choices. (page 8)

[T]he potential risks to privacy from care.data are:

A. Loss of individual autonomy from use of patient identifiable data without consent

B. Risk of confidential information being accessed and viewed without knowledge or consent of patients

C. Linking and de-identification processes may not be reliable enough to achieve total anonymisation of data

D. Risk of data being accessed illegally and then sold or otherwise misused by commercial organisations, criminals or others; and

E. Risk of data being accessed legally and then the data being misused. (page 15)

[T]he processing of a person’s information without their permission can be considered a loss of autonomy for that individual. (page 23)

The second reason why the care.data Privacy Impact Assessment produced by NHS England is relevant to our discussion, has to do with what the supposed ‘opt-out’ model of the care.data scheme really means. Despite containing assurances to the contrary, the care.data Privacy Impact Assessment makes it clear that patients registering their wish to opt out of the scheme to make their medical information available for research, *will not have those wishes respected*. The Assessment contains such reassuring statements as:

To mitigate against the risk [that patients may lose trust in the confidential nature of the health service], the NHS constitution gives patients the right to object to their personal confidential data *leaving their GP practice*. In line with the commitment given by the Secretary of State for Health in April 2013, *patient objections will be upheld* other than in exceptional circumstances such as a public health emergency. (page 6, emphasis added)

Patients can object to the processing of the personal confidential data in GP records. (page 8)

Put simply, patients *who are concerned about their privacy* can now control the flow of confidential data both out of their GP practices and out of the HSCIC. (page 13, emphasis added)

The HSCIC and NHS England will respect the wishes of patients who request that their data are not used by care.data, unless there is a statutory duty or an overriding public interest (e.g. public health emergency) to do otherwise… (page 21)

However, despite these assurances, the Assessment makes it clear that the GP data of those registering an opt-out *will* be passed to the HSCIC and will most likely be used in research to which those patients have not consented. Thus the Assessment reports that:

Where patients have objected to the flow of their personal confidential data from the general practice record, the HSCIC will receive clinical data without any identifiers attached (i.e., anoymised data). (page 9)

More particularly, the Assessment continues:

If a patient is (a) content for personal confidential data from their GP record to be extracted into the secure environment of the HSCIC but (b) objects to flows of personal confidential data from the HSCIC … then the HSCIC will extract the fact of the objection, the date of the objection and the individual’s NHS number. The NHS number will be used internally within the HSCIC to match these data to other data held for that patient *so that the data can be anonymised before release*. (page 10, emphasis added)

In this context, by ‘anonymisation’ is in fact meant ‘pseudonymisation’, a ‘technique that replaces identifiers with a pseudonym that uniquely identifies a person’ (page 31), i.e. what is frequently called ‘coding’ of health data. Astonishingly, it appears that the NHS’s understanding is that a patient’s wish that her confidential information is *not* extracted or used, is respected by extracting and using the data in pseudonymised form. This would undoubtedly come as a surprise to most if not all citizens who have opted out of the care.data scheme and therefore think that their data *will not be used in any way*. We will come back to this when making some recommendations below.

## The difficult balancing of various interests

The discussions around the implementation of the care.data scheme and the concerns raised by various professional medical bodies and citizens illustrate the challenges involved in finding a balance between on the one hand aiming to improve the quality of care and health services (as well as stimulating research) and on the other hand respecting ethical values such as trust, respect for autonomy, transparency, and respect for confidentiality and privacy.

Trust is critical in determining whether individuals will support a programme such as the care.data scheme. Public trust and public support are complex phenomena and various factors play a role in the level of trust individuals have in healthcare organizations, governmental entities and research institutions.^30^ The various issues illustrated above with numerous quotes show real concerns that exist among citizens. These concerns should not be taken lightly by anyone who finds public trust in such institutions important.

As argued by bioethicist Julian Savulescu with regard to the use of leftover body material: “Each mature person should be the author of his or her own life. Each person has values, plans, aspirations, and feelings about how that life should go. People have values which may collide with research goals … To ask a person’s permission to do something to that person is to involve her actively and to give her the opportunity to make the project a part of her plans. When we involve people in our projects without their consent we use them as a means to our own ends.”^31^ This is essential and it is one of the main reasons why it really matters to study citizens’ concerns regarding care.data and to take their concerns seriously. This is important not only from a sociological or political point of view but also from an ethical point of view. Even if we put aside the issue of whether the use of people as a means to an end could be morally justified in some particular situations, there is no doubt that people do not like to be used as means to an end without their consent. They can be either coerced into it, or manipulated, or both, but the point is that all such options are resented.

It is clearly the manipulative element that some people perceive in the care.data scheme that violates trust. Trust is however essential for making the whole scheme work. This is even more so because the entire scheme revolves around health data. Indeed, in order to merit any trust and to be trusted, those who acquire health data ought to make sure that they respect the autonomy of individuals who are expected to *entrust* them with their personal data.^32^

The principle of respect for autonomy is based on the principle of respect for persons.^33^ Taking these principles seriously implies that people should be offered appropriate ways to (not) consent to have their health records included in the central database, which in turn implies that transparency is a crucial prerequisite. Moreover, not only to avoid violations of the principle of respect for autonomy, but also in order to earn and obtain citizens’ trust, it is essential that the whole care.data scheme is transparent. Transparency is key to trust and trust is key to making care.data work. As we have seen, however, various criticisms are connected to the lack of adequate information and transparency with regard to the implementation of the care.data scheme. This shows that prior consultation and communication with local communities and interests groups is crucial in contexts such as this.^34^ Participation of the public may create more representative and accountable policies, thereby ensuring a larger societal support for the policies in question, which should not only reflect the perspectives of medical professionals, academics and politicians.^35^ The various criticisms expressed by citizens as discussed above show the feeling that citizens have not been taken seriously, either in the designing of the care.data scheme, or as regards their right to decide whether or not to participate in the scheme.

The issue of informed consent is certainly one of the most controversial topics in the context of health care and research. The various quotes above not only demonstrate a lack of information regarding the care.data scheme, but also show that citizens were denied decisional capacity. Various citizens emphasised that a program such as care.data should be on an ‘opt-in’ basis or at the very least on a transparent and straightforward ‘opt-out’ basis.^36^ The comments also highlight the importance these citizens attach to being informed and being given the opportunity to approve the use of their personal health data, to know who outside the NHS would be using the data and for what purposes.

Various criticisms were made regarding the potential commercialisation of personal health data. Commercialisation in this context could take at least two different forms. First, where the HSCIC *would be used as a profit source by the UK government*. In this regard, the HSCIC reassures people that it will not make a profit from providing data to other organizations, but will only charge an access fee to cover its costs.^37^ While this may look unproblematic, it in fact means that commercial companies have access to assets they have not themselves bought or created and are thus being given a quasi-free commercial boost by the UK government. To put NHS databases at the disposal of industry, without requiring a ‘kick-back’ to enhance the service that the NHS is set up to provide, is inappropriate. A more just arrangement would need some form of benefit-sharing, with benefit effectively passing back to the UK citizenry. The mere fact that a new drug might reach the market is not sufficient since this is true for citizens of all other countries, whose health data has not been mined by the companies in question. Instead, the companies seeking access could be required to provide the NHS with reduced access costs for the resulting drugs or other health-related products.

A second form commercialisation could take is that the entities who are given access to health data may *themselves* use it for commercial purposes, e.g. pharmaceutical companies using the data for R&D of drugs which will be sold for profit. The quotes discussed above clearly show that some citizens feel strongly uncomfortable about the fact that the care.data scheme allows commercial companies to have access to their data. According to various international recommendations, researchers should inform research subjects about potential commercial uses of their biological samples and data.^38^ These recommendations are partly inspired by the fact that various studies indicate that people may consider commercial uses to be at odds with their original motivation to participate in research (and this is the case even when they explicitly agreed to take part in research).^39^ Indeed, transparency regarding the potential commercial uses of personal health information is warranted.

Research has shown that the general public is generally positive towards medical research and is usually willing to participate without expecting any personal benefit.^40^ The willingness to participate decreases however if the benefits to society are unclear or if private profits might be derived.^41^

In this context it might also be useful to refer to the ongoing debates on newborn bloodspot cards that are collected in the framework of newborn screening. These bloodspots represent a unique resource for biomedical research and public health surveillance and constitute a valuable resource for a better understanding of the role played by the environment in the development of common diseases, and would allow comparisons between children growing up in different environments. However, recent lawsuits about sample storage and lack of parental consent for the storage of the samples and for their use in research have had a negative spillover effect on the presumed consent basis of newborn bloodspot screening itself and have led to the destruction of valuable collections of bloodspot cards. Driven by patient advocacy groups, various lawsuits have taken place over the last years in Texas and Minnesota. For example, in Texas, five families sued the Texas Department of Health Services for storing bloodspot cards indefinitely and using them for undisclosed research purposes without parental permission. In response to the lawsuit, the Texas newborn screening laws were changed to authorise the retention of the samples, and the lawsuit was settled. In the negotiated settlement, Texas agreed to destroy five million samples that had been retained without parental consent before the new legislation took effect.^42^

Even though the US Office for Human Research Protections holds the position that research using only de-identified materials falls outside the definition of “human subjects research”, thus allowing the use of anonymised archival materials for research without consent, the use of stored newborn blood spots for research has stirred up much controversy and, as mentioned, even litigation in the US. Contrary to what some might argue, anonymisation and pseudonymisation (or coding) of health data does not overcome all objections and concerns that individuals have.^43^ Controversies such as the one surrounding newborn bloodspot cards might be instructive for those responsible for designing programmes such as the care.data scheme. They demonstrate the importance of providing information, transparency, guaranteeing individuals’ right not to participate, and preserving trust in the healthcare system and its associated research programmes.

## Concluding remarks

The concerns expressed by citizens regarding the care.data scheme turn out to be remarkably similar to the concerns expressed by the medical community. Moreover, not only concerned citizens and the medical community have identified these major flaws of the scheme: as is clear from the Privacy Impact Assessment discussed above, *NHS England itself is fully aware that the care.data scheme infringes on personal autonomy*, even though this is the guiding principle behind the requirement for consent, itself a foundation stone for modern medicine and research.

Our most important recommendations for redesigning the scheme would be the following. First, much more transparency and clarity need to be created regarding the existence of the scheme as well as its goals and implications. As discussed earlier in the section providing the background to the care.data scheme, the meaning and boundaries of the amendments made to the Health and Social Care Act remain unclear, for example as regards the kinds of third parties to whom access to the data will be sold and for what kinds of purposes. This makes it impossible for citizens to make an informed decision as to whether they want their health records to be transferred to the database.

Our second recommendation would be that, if selling access to the database to third parties remains part of the scheme (and we have no reason to think that this is being rethought), the UK government and the NHS should require a kick-back from industry. As explained earlier, the HSCIC has declared that it will only charge third parties an access fee to cover its costs. This implies that commercial companies will be given very cheap access to assets they have not themselves bought or created and thus receive a quasi-free commercial boost by the UK government. However, to put NHS databases at the disposal of industry, without requiring a ‘kick-back’ to enhance the service that the NHS is set up to provide, is clearly inappropriate. Some form of benefit-sharing is necessary, with benefit effectively passing back to the UK citizenry. For example, the companies seeking access could at least be required to provide the NHS with reduced access costs for drugs or other health-related products resulting from R&D that relied (among other sources) on information obtained from the HSCIC.

Our third recommendation is that the scheme should be on an ‘opt-out’ basis. An ‘opt-out’ or presumed consent system is not necessarily ethically problematic. Although opt-in or explicit consent is clearly the default option when research involves humans (and/or their body material and related data), when potential risks are minimal and potential benefits are huge, exceptions to this rule can be allowable. As to risks, in its current form, care.data clearly involves far more than minimal risks. Whether these risks can be reduced to minimal risks will depend on whether or not the necessary changes will be made to the scheme. *If and when that is done*, a presumed consent model could be justified for care.data on the basis of its potential benefits. Indeed, there is no doubt that bringing together, linking and sharing large collections of health data has significant potential benefits. It holds great promise for the development of diagnostic, therapeutic and disease-preventing strategies. The discussion in this paper clearly shows that these potential benefits are acknowledged not only by the medical community but also by many concerned citizens. Hence, provided that the first and second recommendations above are met, an ethical case could be made for grounding the scheme on an opt-out rather than an opt-in (i.e. explicit consent) basis. Indeed, in an entirely different context (i.e. the case of post mortem organ removal) it has been pointed out, rightly in our view, that a presumed consent system does not in any way restrict a person’s right to self-determination, as long as the person is aware of the nature of the system, is aware of the specific implications of not opting out and those of opting out, is allowed a reasonable time period in which to opt out, and is offered adequate and straightforward means of formally recording their opt-out.^44^

However, none of these conditions appear to be met in the case of the care.data scheme, *hence consent simply cannot be presumed*. Moreover, alarmingly, *the scheme is not even in fact based on an opt*-*out regime*, since a patient’s wish that her confidential information is *not* extracted or used, is met by extracting and using the data in pseudonymised form. This is clear from the Privacy Impact Assessments discussed earlier. Moreover, the HSCIC itself makes this crystal clear. In its responses to Frequently Asked Questions, it provides the following answer to the question “Can I stop information that does not identify me being used?”:

No. Information that does not identify you is neither personal nor private and the law says that it can therefore be used much more freely.^45^

This makes a mockery of the reassurance by the UK government that citizens can opt out of the care.data scheme. If somebody opts out, that should mean that their data are simply *not extracted and used*, i.e. HSCIC should receive no data, whether in non-identifiable (pseudonymised or coded) form (i.e. still uniquely identifying the person) or in anonymised form (i.e. not identifying the person).

Again, we wish to emphasise that we have no problem with an opt-out system for this kind of project. Our criticism concerns precisely the fact that care.data is not based on an opt-out but instead boils down to conscription. This is unacceptable, for what is at issue here are people’s health records, i.e. a lot is at stake. An opt-out model represents what could be called the ‘ethical minimum’, for, as explained in this paper, the care.data scheme may involve serious infringements upon the privacy, autonomy, and moral integrity of NHS patients. Indeed, the health data may be used in a way that is incompatible with the moral values of the patient concerned. Following Ronald Dworkin’s (1993) terminology, so-called ‘critical interests’ may be at stake.^46^ Such interests are bound in the projects, plans and choices that persons have made and that give meaning to their life. It is important for the individual that others respect these and do not take actions that will critically impact on them in a negative way. From this perspective, people are entitled to their health data being used in a manner that corresponds to their life story and ethical values. As the US National Bioethics Advisory Commission already observed with regard to human body material in 1999, anonymization (of the material or data) cannot invalidate this claim.^47^ Failure to respect this would amount to using people as a means to an end they have not chosen, i.e. instrumentalization.^48^

Clearly, nothing valuable can be achieved if citizens believe that they are purposefully ill-informed with the aim of disguising various types of problematic practices. It follows from the reactions to care.data of citizens and medical professionals discussed above, that, in order to earn and retain citizens’ trust, the specific concerns identified in this paper need to be addressed in the light of the common values that citizens wish to promote, including the value of furthering the common good. These values need to be furthered on the basis of sufficient and transparent information and an absence of misleading practices of any sort.

As we have argued, the essential starting point is trust. In order to merit any trust and to be trusted, those who are the guardians of citizens’ health data ought to make sure that they respect the autonomy of the people who are expected to trust them with that data. NHS patients should not have any fear that they are being manipulated into sharing their health data, i.e. that they are being used as a means to an end. It is precisely this starting point of trust that has been overlooked from the outset in the design of the care.data scheme. It is to be hoped that the concerns discussed in this paper will be dealt with before a truly transparent and trustworthy version of the scheme is launched.

## Footnotes

1 Meanwhile, members of the Conservative Party seem to have admitted that passing the Act was a mistake. See Palmer (2014).

2 The negative effects of the Act on the NHS, and in particular the negative effects of the relaxation of the obligations placed on the Secretary of State for Health, have been commented upon at length in Davis and Raymond (2013).

3 Department of Health (2011).

4 See Sterckx and Cockbain (2014).

5 http://www.england.nhs.uk/ourwork/tsd/care-data/better-care/.

6 http://bma.org.uk/search?query=Care.data%20-%20Joint%20statement.

7 http://www.rcgp.org.uk/news/2014/december/rcgp-response-to-care-data-report.aspx.

8 Partridge (2014).

9 Indeed, the pressure group medConfidential reported in its Bulletin of 5 September 2014 that ‘Minutes published by the revived Data Access Advisory Group (DAGG) at HSCIC earlier this week revealed that an unnamed organisation has been using HES and ONS data “for commercial activity in addition to the purposes they had stated when applying for approval”.’ See https://medconfidential.org/2014/medconfidential-bulletin-5-september-2014.

10 Apparently, in the ‘pathfinder’ areas ‘there will be lots of activity: local media work, and a new set of communication materials, including a letter sent by name to every patient, will be used to raise awareness of the programme and support GPs prior to a period of data extraction in those areas only in early 2015.’ See http://www.healthwatchwalthamforest.co.uk/news/caredata-oct-2014.

11 See medConfidential Bulletin (2015) https://medconfidential.org/2015/medconfidential-bulletin-1-june-2015.

12 See medConfidential Bulletin, 5 September 2014, above, note 9.

13 Matthews-King (2014).

14 http://bma.org.uk/practical-support-at-work/ethics/confidentiality-and-health-records/care-data.

15 http://www.england.nhs.uk/ourwork/tsd/care-data/gp-guidance/.

16 Monaghan (2014) see: https://iconewsblog.wordpress.com/2014/01/27/ico-blog-nhs-patient-information-and-the-data-protection-act/.

17 Grace and Taylor (2013).

18 Grace and Taylor, above, note 17.

19 Grace and Taylor, above, note 17, at 430.

20 Grace and Taylor, above, note 17, at 432 (footnotes omitted).

21 Grace and Taylor, above, note 17, at 435–436 (emphasis added).

22 See http://www.hscic.gov.uk/media/12931/Privacy-Impact-Assessment/pdf/privacy_impact_assessment_2013.pdf.

23 See http://www.england.nhs.uk/wp-content/uploads/2014/01/pia-care-data.pdf. We thank Dr Edgar Whitley for bringing this to our attention.

24 http://www.nhs.uk/NHSEngland/thenhs/records/healthrecords/Pages/care-data.aspx.

25 Of these, the first 189 were analysed in the initial study for this paper. The remaining 12 were analysed during the revision and review process of the paper, but were found to contain no additional points of concern.

26 Braun and Clarke (2006).

27 The blogs relate to the article ‘NHS patient data audit uncovers ‘significant lapses’ in confidentiality’, 17 June 2014 (available at http://www.theguardian.com/society/2014/jun/17/nhs-patient-data-audit-significant-lapses-confidentiality-hscic [last accessed 1 June 2015]), as well as the article ‘How care.data could help save lives’, 18 August 2014 (available at http://www.theguardian.com/healthcare-network/2014/aug/18/care-data-confidentiality-help-save-lives-health-nhs [last accessed 1 June 2015]). The authors of the second article are employed by a data customer of a forerunner of the HSCIC.

28 HSCIC, above, note 22.

29 NHS England, above, note 23.

30 Resnik (2011).

31 Savulescu (2000).

32 In this regard, it is interesting to note that the citizens’ concerns discussed above show many interesting similarities with concerns raised in the academic literature on health data privacy. See for example Lowrance (2012), Taylor (2011), Lane and Schur (2010), Bobrow (2013).

33 For an accessible explanation, see National Commission for the Protection of Human Subjects of Biomedical and Behavioral Research (1979).

34 Morone and Kilbreth (2003).

35 Litva et al. (2002).

36 The point might be raised that allowing opting-out, or requiring opting-in, might result in an undesirable selection bias in the care.data data set. This is indeed correct. However, such selection bias may readily be adjusted for by random deselection of data sets from over-represented patient categories, e.g. gender, age, location, condition, etc. Selection bias is thus not an adequate reason for conscription of patient data.

37 See http://www.nhs.uk/NHSEngland/thenhs/records/healthrecords/Documents/FAQs-for-patients-vs5.pdf.

38 HUGO Ethics Committee (2002), Organization for Economic Co-Operation and Development (2009), Canadian Institutes of Health Research et al. (2010), European Society of Human Genetics (2003).

39 Skolbekken et al. (2005), Godard et al. (2007), Nilstun and Hermerén (2006).

40 Nicol and Critchley (2012).

41 Critchley et al. (2012), Kettis-Lindblad et al. (2006).

42 Lewis et al. (2012), Hu (2012).

43 Van Assche et al. (2013).

44 Den Hartogh (2008).

45 NHS England, above, note 37.

46 ‘Critical interests’ need to be distinguished from so-called ‘experiential interests’ which are related to the pursuit of pleasurable experiences. Contrary to ‘critical interests’, the setback of experiential interests will be temporarily frustrating at most. See Dworkin (1993).

47 “It is incorrect to assume that because the sources cannot be identified they cannot be harmed or wronged. […] Individuals have an interest in avoiding uses of their tissues they regard as morally impermissible or objectionable. Thus, were their materials to be used in research that they would consider objectionable, it is possible that some individuals could be wronged, if not harmed”. See National Bioethics Advisory Commission (1999). At most, anonymization might offer protection with regard to privacy, although several recent studies suggest that even this cannot be guaranteed. See for example McGuire and Gibbs (2006).

48 We are not suggesting here that it is *never* permissible to do something to a competent person that does not correspond to their life story and ethical values. In exceptional cases coercion might be permissible (e.g. mandatory immunization; coerced placement and treatment) but these interventions find their justification in averting a *grave* and *direct* danger to the person concerned, third parties or society at large. These conditions do not apply to the context at issue here (care.data).
